# When Public Health Goes Wrong: Toward a New Concept of Public Health Error

**DOI:** 10.1017/jme.2023.67

**Published:** 2023

**Authors:** Itai Bavli

**Affiliations:** 1. UNIVERSITY OF BRITISH COLUMBIA, VANCOUVER, CANADA.

**Keywords:** Public Health Error, Public Health Failure, Public Health Ethics, Opioid Epidemic, COVID-19

## Abstract

Studies of public health decisions that have had harmful effects tend to disagree about what constitutes a public health error. Debates exist about whether public health errors must be culpable or not, as well as about what the criteria for judging public health errors should be.

## 1. Introduction

In an October 2020 opinion article on CNN,^1^ David Holtgrave, Dean of the University of Albany School of Public Health, suggested that the Trump administration committed multiple public health errors in response to the COVID-19 pandemic. Holtgrave outlined twelve possible errors made by the administration that harmed the United States’ national COVID-19 response, including failing to develop initial testing strategies; discouraging states from adopting effective strategies such as physical distancing, mask use, and avoidance of large gatherings; and more. These failures, according to Holtgrave, may have been avoided if the Trump administration was using evidence-based policies in its response to the virus, which in turn would have prevented unnecessary deaths of Americans. The notion that a government can commit public health errors in its response to a public health emergency like the virus — and that these errors can negatively impact a large number of people — has started to receive attention from the scientific community and the popular press. However, despite this growing interest in public health errors, the concept of public health error is not fully understood, the phrase lacks an agreed-upon definition, and little research has been done on the topic.

One of the most important goals of medicine is to ensure that the benefit of a medical intervention outweighs any harm. Physicians should balance their obligation to benefit the patient (the principle of beneficence) against not causing harm (the principle of non-maleficence).^2^ If physicians neglect to protect their vulnerable patients, they fail to fulfill their duty. Nevertheless, medical errors happen and represent a significant health threat to many patients, leading to the deaths of tens of thousands in the United States each year.^3^ The U.S. Institute of Medicine (IOM) defines an error as, “[T]he failure of a planned action to be completed as intended (error of execution) or the use of a wrong plan to achieve an aim (error of planning). An error may be an act of commission or an act of omission.”^4^

Medical errors, such as when a surgeon makes an incision in the wrong place, are widely studied and discussed,^5^ yet very little attention has been given to public health errors — actions (or inactions) of public health systems that can affect an entire population.^6^ Studies on policy errors (including regulatory errors),^7^ the harmful effects of some public health interventions,^8^ and public health errors^9^ have treated errors in various ways.^10^ While there is a general agreement in the literature that errors can be of action or omission, disagreement exists about whether culpability should be regarded as an inherent feature of public health errors.^11^

Furthermore, in the related literature on policy failure,^12^ there is a debate about what the criteria for judging policy failures should be (e.g., is it whether or not the public good was promoted, or whether or not political goals were achieved?). Last, while a negative outcome of a policy choice is often referred to as an indication of an error,^13^ it is still unclear how public health errors differ from the adverse effects of public health interventions.

To address these issues, I have described a newly developed concept of public health error. I define a public health error as an action or omission by public health officials whose consequences for population health are substantially worse than those of an alternative that could be chosen instead. This definition implies two things: that both culpable and non-culpable errors should be considered as public health errors, and that a different decision would have enabled more people to have lived longer or been healthier. My proposed definition of public health errors includes interventions that directly caused harm to the health of the population and were worse than doing nothing at all; failure to take action when measures were needed to protect the health of the population; and finally, interventions that did some good but which were still significantly inadequate in comparison to available alternatives.

In the first part of the paper, I review the literature on public health errors and related works on policy errors, policy failures, and the harmful effects of public health interventions. In the second part, I present my conception of public health errors and explain its contrasts with previous approaches. In the section that follows, I present the main advantages of the new conception. Last, I discuss three case studies of public health errors: radiation treatment, the opioid epidemic in the US, and the opioid epidemic in Canada. I will demonstrate how these three examples represent different types of public health errors, and how the proposed concept of public health error helps better assess them.

This conception has several advantages over previous attempts to define public health errors. First, by focusing on health outcomes, the proposed concept better aligns with the aim of public health, which is to maintain and promote the health of populations,^14^ compared to policy failure literature, where achievement of political objectives is often used to measure success. In this sense, public health errors of action or omission are contrary to this aim (causing or failing to prevent harm to the public). Second, not making culpability part of the definition better corresponds with common usage and makes it easier to decide whether an action should be counted as a public health error. Last, public health errors often have similar causes and effects, whether they are culpable or not and whether they are by action or omission. Thus, it is theoretically better to have a public health error concept that does not require culpability, because scientific categories should identify classes of things or events that have similar causes and effects.^15^

By focusing on the consequences of public health choices rather than on culpability — and acknowledging errors of omission and commission — the conception allows for the investigation of a variety of public health choices that lead to similar results. For example, in the field of drug regulation, approving a faulty drug is considered an error of commission, while the failure of regulators to adequately respond to evidence of adverse effects is an error of omission. And these errors may be culpable or not. Yet in any of these cases, harms to patient health ensue. In addition, further consequences can follow, such as loss of trust in health authorities, and these consequences can be shaped by social inequalities whether the error was culpable or not.^16^ The proposed concept also permits the consideration of mechanisms leading to public health errors, regardless of whether the error was an action or omission or culpable or not.

The concept of public health error proposed here contributes to and extend existing studies on the various harmful causes and effects of public health interventions,^17^ studies that have sought to identify and define erroneous public health decisions,^18^ and studies on regulatory errors and policy failures.^19^ The current work provides a clearer and more justified conception of public health errors, discusses several cases of public health errors, highlights some of the challenges in identifying such errors using multiple current and historical examples, and provides some practical recommendations based on key lessons learned from these case studies.

In the first part of the paper, I review the literature on public health errors and related works on policy errors, policy failures, and the harmful effects of public health interventions. In the second part, I present my conception of public health errors and explain its contrasts with previous approaches. In the section that follows, I present the main advantages of the new conception. Last, I discuss three case studies of public health errors: radiation treatment, the opioid epidemic in the US, and the opioid epidemic in Canada. I will demonstrate how these three examples represent different types of public health errors, and how the proposed concept of public health error helps better assess them.

## 2. Review of Previous Work

The concept of public health errors should be considered in connection to a broader understanding of the aims and scope of public health. Public health, according to the IOM, is “what we, as a society, do collectively to assure the conditions for people to be healthy.”^20^ Unlike clinical medicine, public health approaches are focused on and directed to communities, populations, and the broader social and environmental influences of health.^21^ A public health system includes all governmental public health agencies, public health and health science academia, and the health delivery system;^22^ public health interventions are the activities of the system and entities that aim to promote and protect the public’s health, for example, the immunization of schoolchildren, engaging in epidemiological research, ensuring a safe water supply, or assessing the safety and efficacy of new drugs.^23^ In this section, I review prior discussions of the concept of public health error found in the literature. Since literature that focuses specifically on public health errors is rather sparse, I also examine literatures on related topics within the same family of concepts, including regulatory error and policy failure.

### 2.1 Prior Attempts to Define Public Health Error

The concept of public error is motivated by the fact that public health interventions can sometimes result in wasted public money, limited improvement in a public health issue, or even harm to the public.^24^ Lorence and Oliver^25^ identify five broad categories of possible adverse effects of public health interventions: direct harms, psychological harms, equity harms, group and social harms, and opportunity cost harms. They emphasize that policymakers should consider these potential side effects when deciding on, implementing, and evaluating specific public health interventions. Building on their framework and acknowledging the relative scarcity of available data on the various adverse effects of public health interventions, Bonell and colleagues^26^ focused not only on potential harmful outcomes but also on identifying mechanisms of harm. They evaluated harm more narrowly, defining harm that affects individuals receiving an intervention and that can be detected (excluding rare side effects), opportunity harm (indirect harm to recipients), and inequalities in intervention benefits.

These authors also split adverse effects of public health interventions into two broad categories: *paradoxical effects* and *harmful externalities*. *Paradoxical effects* refers to interventions that increase the adverse outcomes they seek to prevent. For example, an educational program delivered to teenagers in England, ages 13–15 years, aimed to reduce teenage pregnancies, drug use, and school exclusions. Evaluations of the program, however, found higher rates of pregnancy among girls in the intervention group than in the control group, as well as more students missing school days.^27^

*Harmful externalities*, on the other hand, refers to public health interventions that produce harms in outcomes that the intervention did not target.^28^ An example is the adverse effects of a drug^29^ that are unrelated to the effect the drug was intended to have. A drug can be effective in relieving pain, for example, but also risky for patients who develop addiction to it (in the case of opioids) or lead to an increased risk of heart attacks (in the case of the painkiller Vioxx).^30^

Recognizing that public health decisions can do harm, public health and policy scholars have tried to identify what should be considered a public health error and explored the factors that can lead to errors. The IOM defines an error as, “[T]he failure of a planned action to be completed as intended (error of execution) or the use of a wrong plan to achieve an aim (error of planning). An error may be an act of commission or an act of omission.”^31^ Building on the IOM definition of error, David Holtgrave defined public health errors as follows: “A public health error occurs when one or more stakeholders in a public health system commit(s) a cognizant, negligent act of commission or omission that fails to achieve necessary public health outcomes.”^32^ He emphasizes that error of omission (i.e., failing to act) is an important and sometimes neglected part of the definition. He also distinguishes between “public health errors” and the adverse *effect* of a public health intervention, focusing on the intent rather than on the outcome for assessing errors. According to Holtgrave, bad outcomes as a result of cognitive and judgment limitations should not be counted as public health errors.

Holtgrave provides three possible categories of public health errors.^33^ The first category, “errors of deliberate commission,” is an act of deviation from the norms, law, and existing standards. This type of error occurs when health officials act with the knowledge of doing something that violates the law and norms when other courses of action are available. The second type of error he describes is “errors of willful omission.” This type of error occurs when health officials know about the necessity of a particular action to improve public health but consciously decide not to take this action. An example of the latter is failure to control exposure to tobacco in public areas, although it is known that exposure to this factor is harmful to people who pass through these public areas. The third and last type of public health errors Holtgrave identifies is “errors of complacency.” In this category, relevant public health actors had the resources and knowledge and should have paid attention to particular public health hazards but failed to do so. An example might be if a public health actor fails to keep up with relevant literature and as a result decides on the wrong intervention; another could be someone paying insufficient attention to a growing public health issue. In all three categories, Holtgrave highlights lack of concern or actual intent to do harm as the most important components for identifying public health errors.

In response to Holtgrave, De Ville and Novick^34^ challenged the idea that intent to do harm is essential to the definition of public health errors. “Errors of deliberate commission,” they argue, should be categorized as unjustified violations or breaches, as opposed to errors. They do not offer a new definition for the concept, but they do agree with Holtgrave that failing to act (error of omission) is an important factor for understanding the range of public health errors. They argue, however, that Holtgrave conflates errors with culpability and suggest that “flawed state-of-the-art errors” should be considered as instances of public health errors. Flawed state-of-the-art errors “might be viewed as those decisions or actions that prove mistaken or incorrect, even when the decision-maker could not have decided or acted in a more advantageous way, given the current state-of-the-art and existing knowledge.”^35^ Hence, they argue that culpability should not be viewed as necessary in defining a public health errors; rather, they claim that there are a range of possible errors that cannot be traced to negligence or other types of culpable actions by health officials.

Remarkably, the exchange between Holtgrave and De Ville and Novick exhausts the literature specifically focused on defining the concept of public health error. Consequently, I will also examine closely related literatures.

### 2.2 Agency Failure and Regulatory Error

The possibility of errors that impact public health are also discussed in the works of scholars who focus on failures of administrative agencies^36^ and regulatory errors.^37^ Heimann, for example, emphasizes two types of agency errors: (1) errors of commission in which the wrong policy is implemented; and (2) errors of omission where there was a failure to act when action was warranted.^38^ He notes that previous studies mostly assumed two possible organizational performances: an agency may adopt the most effective policy to achieve the program goals or adopt an ineffective policy that does not achieve the program goals. This literature, Heimann suggests, ignored the fact that agencies can fail to act.^39^

Building on Heimann’s recognition of the possibility of two types of errors,^40^ Carpenter and Ting,^41^ in investigating the FDA’s behavior, discussed factors influencing regulatory errors (i.e., errors made by the FDA).^42^ In their first study (published in 2005),^43^ they associate errors with some degree of culpability, similar to Holtgrave,^44^ and focus on the information the FDA had at the time of approval. They argue that a drug withdrawal by the FDA (when the agency determines that a drug’s benefits no longer outweigh its risk) is not necessarily a result of a regulatory error if the adverse effects could not have been detected in the approval process and if the regulator appropriately acts to detect such adverse effects after approval. They noted: “…some drug-related adverse events^45^ might be so uncommon that they could not have been detected in the clinical trial phase of the approval process, and if the regulators act promptly on detection of such events after approval and use in a larger population, it might be argued that no error has been made.”^46^

By focusing also on how the FDA responds to the discovery of adverse effects, Carpenter and Ting^47^ added a new layer to the discussion. In the area of drug regulation, response to adverse effects or errors is known as post-market regulation. It includes all the activities taken by the health regulator to monitor drugs for safety problems after marketing, including updating label claims and removing a drug from the market.^48^ By merging two distinct categories into one broad one, they highlight uncertainties regarding what is and is not an error. As they noted: “…we, therefore, leave the necessary medical and public-health judgment [whether their cases represent errors] to others.”^49^

In their second study (2007), Carpenter and Ting^50^ elided their previous comments about the link between errors and responses to errors or issues of culpability, focusing instead only on the outcomes of the FDA’s regulatory decisions (approving bad products and rejecting good products).^51^ According to Carpenter and Ting, to indicate that an error was made, “the FDA must attach evidence of new contraindications, or new side effects, that are serious in some way,”^52^ or international regulators must remove the drug from the market. In both studies, uncertainties remain regarding what constitutes an error. In their first study,^53^ Carpenter and Ting focus on culpability and responses to decisions that have had harmful effects. The second study^54^ measures outcomes, signaling that an error was made. Further, the question remains why significant label revisions should be considered evidence of an error as opposed to, for example, less significant label revisions or adverse effects associated with a drug.

### 2.3 Policy Failures

A related field of study on the subject of policy failures examines cases of policies that have failed in different policy areas and across regions.^55^ This literature highlights the variety of policy failures and raises questions of what constitutes a policy failure (e.g., what the standard should be for judging errors).^56^ Studies in this literature examine, for example, factors leading to policy failures;^57^ the variety and extent of government failures;^58^ the link between policy learning and policy failures;^59^ and the negative political consequences and blame associated with failures.^60^ Most of the studies in this literature examine policy failures across the *process*, *program*, and *politics* of the policy cycle.^61^

Policy failures are often treated as political failures, such as a failure to gain political approval of a specific policy initiative, failure to achieve political support after implementation, or decline in political or agency legitimacy.^62^ Thus it is possible for there to be multiple errors during the political process. McConnell^63^ highlights the possibility of degrees of failures (e.g., a program can partially fail or succeed to achieve goals) and demonstrates that there is sometimes tension between failure and success; policies can succeed in one aspect of policy-making (e.g., in implementing a policy) but fail in another (e.g., failing to achieve political support after implantation). He also highlights the difficulty of measuring outcomes against multiple and sometimes hidden political goals and in relation to different benchmarks; for example, failure to meet the original objective, to be implemented as intended, to benefit the intended target group, to garner support from key stakeholders, or to provide benefits that outweigh the risk.^64^ A policy fails, according to McConnell, “even if it is successful in some minimal respects, if it does not fundamentally achieve the goals that proponents set out to achieve, and opposition is great and/or support is virtually non-existent.”^65^

Similar to Holtgrave’s notion of public health errors^66^ and Carpenter and Ting’s idea of regulatory errors,^67^ the allocation of blame (and holding the government accountable for the damage done) is often central to the analysis of policy failures.^68^ An example would be when there is an avoidable and blameworthy failure to see the problem developing, or when someone deliberately ignores a problem.^69^ However, as Howlett^70^ recognizes, in some cases, rigorous analysis and execution can still lead to a failure to achieve goals. Rachlinski and Farina,^71^ similar to public health scholars,^72^ acknowledge that good intentions can fail to accomplish a planned goal. They argue that the focus should be on fallibility (e.g., flawed human judgment and choice among policymakers) rather than culpability when studying policy errors.

Three primary observations can be drawn from the literature reviewed above: (a) there is a general recognition that public health errors as well as regulatory or policy failures can be omissions as well as actions; (b) there is disagreement in the literature about whether public health errors and regulatory and policy failures must be culpable; and (c) in the literature on policy failure, there is debate about what the criteria for judging policy failure should be (e.g., is it the achievement of a political objective or the promotion of public good? Is it a partial success or failure?). In the next section, I will address these issues and develop a new concept of public health error.

## 3. A New Concept of Public Health Error

In this section I provide a new definition for the concept of a public health error. My definition builds on previous literature that emphasizes the variety of harmful effects of public health interventions,^73^ adds clarity regarding the disagreements about what represents a public health error,^74^ and how it is different from a policy failure.^75^ Acknowledging the variety of errors (e.g., errors of commission and omission, blameworthy or not), I suggest four broad categories of public health errors: culpable errors of commission, non-culpable errors of commission, culpable errors of omission, and non-culpable error of omission. In line with Bonell et al.’s^76^ notion of *paradoxical effects* and *harmful externalities*, interventions that increase the adverse outcomes they seek to prevent and those that produce harm through other outcomes will both be considered public health errors.

I define a public health error as an action or omission, by public health officials, whose consequences for population health were substantially worse than those of an alternative that could have been chosen, regardless of the causal processes involved in the consequences. This definition suggests that a decision is a public health error when a different decision would have enabled more people to have lived longer or been healthier. It also implies that both culpable errors and non-culpable errors should be considered as public health errors.

By *public health officials*, I refer to everybody who has the power to make decisions about the public’s health, including government regulators, health administrators, politicians, and other stakeholders in a public health system.^77^ By “substantially worse” consequences than an alternative that could have been chosen (i.e., when a different available decision would have enabled people to have been healthier), I refer to the outcome of choosing a worse policy compared to other available options. Measures that were only slightly suboptimal are not counted as public health errors by this definition because they are not *substantially* worse. *Substantially*, therefore, should be understood as entailing a significant degree of error. A public health error occurs when a public health choice turns out to be substantially worst in retrospect (i.e., a better option could have been chosen). This decision must either cause direct and significantly greater harm to the public or fail to effectively prevent harm, compared to other available options.

In some cases — what I call *grey areas* — it would be hard to identify public health errors because in retrospect it is either unclear and debatable whether a different intervention could have produced better health outcomes, or because evaluating the outcome is based on one’s ethical views. For example, consider the ethical problem in public health of the potential tradeoff between efficiency (aggregate health) and equity (inequality of health). A public health intervention can improve the aggregate health but also increase inequality in health (e.g., improves the health of wealthy people, but not poorer people).^78^ In these cases, it might be unclear whether an error was made because of ethical considerations and how one’s values lead one to weigh different principles.^79^ Only cases in which the ethical arguments clearly favor one option over another will be considered as public health errors.

Consider the discussion around measures to contain the COVID-19 virus. Stricter economic lockdowns and isolation measures can improve the average health (reducing the viral spread and the associated harm), but for poor or other vulnerable populations, who depend on their daily income to survive (especially when no government support is provided), such measures can cause severe harm, including deaths.^80^ This increases inequalities in health. In the case of COVID-19, some actions (e.g., closing overdose prevention sites) and omissions (e.g., failure to take any measures to prevent the spread of COVID-19 in long-term care facilities) would be public health errors in the sense defined here. However, there could also be a range of policies for which it would be inherently debatable which was best, and consequently no choice among that reasonable suite of policies would count as a public health error.

Public health errors also include cases where a public health decision does some good, but a different choice could have led to a much better outcome for the public’s health. For example, Health Canada’s inadequate and delayed revision of misleading information that appeared in the OxyContin product monograph^81^ is considered as an error because it failed to prevent a much greater harm, although the monograph change did some good (updating and revising some misleading claims). A different action, such as more quickly deleting the misleading information and warning physicians of the danger, could have prevented the drug manufacturer from using this information in its promotional practices and, in turn, saved lives (see section 5.2).

Based on the recognition above that a public health decision can do some good but still be considered a public health error, doing more harm than good will not be considered here as a necessary condition for a public health error to occur. Some errors cause harms greater than the benefits (e.g., the adverse effects of the drug Thalidomide),^82^ while others will not (e.g., insufficient actions taken to control tobacco).^83^ Situations of the latter type are common in errors of omission, wherein the error did not consist of directly causing harm but rather in not doing enough to prevent it. The idea that we should evaluate the outcome of a public health decision in relation to alternative choices is a key component of the definition of public health errors proposed here.^84^

In sum, the definition of public health errors proposed here includes interventions that directly caused harm to population health and were worse than doing nothing at all; failure to take action when measures were needed to protect the health of the population; and finally, interventions that did some good but which were still significantly inadequate in comparison to alternatives. Meanwhile, measures that were only slightly suboptimal would not be counted as public health errors because they are not substantially worse. Similarly, grey area cases, in which judgments about which intervention is best, depend on debatable ethical premises, would not be counted as public health errors because the chosen option is not clearly worse than the alternative.

The above conception has several advantages over previous attempts to conceptualize public health decisions that have been wrong. I present here the conception’s main benefits: (1) focusing on population-level health outcomes better corresponds to the task of public health; (2) not including culpability as a necessary component of public health error results in a definition that is more in keeping with common usage and easier to apply in practice; and (3) the new concept of public health error permits the consideration of general mechanisms leading to public health errors that can be relevant for actions and omissions as well as culpable and non-culpable errors.

Given the definition of public health errors I purpose here, public health errors can be culpable or not, and by action or omission. Culpable errors describe cases in which public health actors took action that, given the information available to them, could have better promoted the public’s health, and the error carries some degree of culpability (e.g., acts of negligence, carelessness, or inattention).^85^ For example, when Thalidomide was approved in Canada, public health officials in Health and Welfare Canada (today, Health Canada) who approved the drug failed to assess the evidence and foresee the risk.^86^ Similarly, the failure to act against the harmful effects of tobacco in the United States,^87^ the delayed action to reduce child poisoning caused by lead paint inside U.S. homes,^88^ and the time it took for government officials to respond the elevated levels of lead found in the drinking water of residences in Flint, Michigan,^89^ can be considered as errors with a degree of culpability. Non-culpable errors (of commission or omission) describe cases in which relevant public health actors took actions that, given the knowledge they had at the time, were expected to produce the best results.^90^ For example, the late health effect of low-dose radiation used for treating benign conditions can be considered as a non-culpable error (I will discuss this example in the last section).

In the next section I will explain this new conception’s major advantages over previous attempts to conceptualize errors.

## 4. The Benefits of the New Concept of Public Health Error

The above conception has several advantages over previous attempts to conceptualize public health decisions that have been wrong. I present here the conception’s main benefits: (1) focusing on population-level health outcomes better corresponds to the task of public health; (2) not including culpability as a necessary component of public health error results in a definition that is more in keeping with common usage and easier to apply in practice; and (3) the new concept of public health error permits the consideration of general mechanisms leading to public health errors that can be relevant for actions and omissions as well as culpable and non-culpable errors.

The first advantage of my conception of errors is that this concept is well suited to the aims of public health. Since the goal of public health is to promote and protect population health and to maintain conditions for people to be healthy,^91^ it is reasonable that this goal figure centrally in a concept of public health error. Thus, approaches in which achievement of political objectives are used to measure success or failure^92^ would not be appropriate for the concept of public health error. What matters from a public health perspective is that interventions should have the overall effect of improving population health, regardless of the political consequences of a certain policy choice. A leader who loses an election due to a politically unpopular decision that produces significant public health gains may have made a political mistake but has not committed a public health error. Of course, analogous observations could be made for other areas of public policy (e.g., enacting a socially beneficial tax policy might be politically risky), but that does not diminish the validity of this point in the context of public health.

In contrast, studies that focus on failure during the policy process (for example, failure to gain authoritative approval of a specific policy initiative or failing to achieve political support after implementation)^93^ highlight the possibility of multiple errors during the policy-making process or the idea that a policy can succeed in one aspect of the policy cycle and fail in another.^94^^.^ However, these policy failures may or may not affect the public’s health. Consequently, these kinds of considerations, though important for understanding why some policies fail, are not directly tied to the task of public health — promoting and protecting the health of populations. Therefore, it is better from a public health perspective to assess public health errors based on population health outcomes, as my conception suggests. That implies that McConnell*’*s framework^95^ about the process, program, and politics important for understanding policy failures — also adopted by others^96^ — does not travel well into the realm of public health errors, where a non-political outcome is most important.

A second advantage of the proposed concept is that it does not regard culpability as a necessary condition for public health errors. Consider, for example, the case of radiation treatment for benign illnesses. Radiation treatment was effective in curing non-life-threatening conditions — mostly benign skin diseases such as acne and ringworm of the scalp.^97^ However, the serious long-term harm for patients who underwent the treatment could be fatal (see section 5.1). This case is commonly regarded as an error,^98^ and since it impacted the health of populations, rather than an individual, it would be a public health error. Yet, the decision to prescribe low-dose radiation therapies for benign diseases was compatible with the best available scientific data at the time^99^ and is therefore non-culpable. Thus, a concept of public health error that does not include culpability as an essential component is more consistent with common usage, which is a practical advantage. Moreover, the radiation example illustrates that, even if non-culpable, public health interventions that generate harmful consequences may have adverse impacts on public trust (see section 5.1).

Making an attribution of blame part of the definition of an error can also make it more difficult to decide whether an action should be counted as a public health error. Consider, for example, Carpenter and Ting’s^100^ focus on culpability when they analyze regulatory errors. Because they focus on blameworthiness, rather than on the outcome, they are unable to determine whether the severe adverse effects (heart attacks, deaths) associated with the pain killer Vioxx was an error or not.^101^ Put differently, focusing on blame can mean that approving a faulty drug might not be considered as an error despite severe harms to patients. Thus, a definition of public health error that does not require culpability is easier to apply in practice.

In sum, the reasons not to limit public health errors to actions also apply to culpability. Like actions and omissions, culpable and non-culpable cases are often difficult to distinguish for empirical as well as philosophical reasons, and the term “error” is commonly applied to both. Given this, it is better not to include these distinctions within a definition of public health error. Once a public health error as defined here has been identified, the role of negligence or other forms of culpability can be investigated.

The third advantage of the conception is theoretical. Scientific categories must share some common properties that play a role in inductive generalization and explanations.^102^ Categories useful to science should pick out a collection of things about which generalizations can be usefully made, and this usually implies some commonality of underlying mechanisms, and consequently some similarity of causes and effects.^103^ Public health errors can have similar causes and effects, whether they are culpable or not and whether they are actions or omissions. That is a further reason to prefer a public health error concept that does not require culpability to one that does.

The suggested concept of public health errors emphasizes mechanisms that can lead to public health errors; generally, these are mechanisms that are not tied to commission or omission, and that apply to culpable or non-culpable errors. A mechanism can be relevant to any of the four combinations (omission, commission, culpable, non-culpable) and cut across these types. There are multiple possibilities of such mechanisms leading to public health errors. They include, for example:

1. Scientific uncertainty: Effects of public health interventions are often difficult to predict because of the inherent complexity of social and economic systems and because of the potential for rare or long-term health impacts,^104^ as in the radiation example. Unintended consequences can result from these uncertainties.
2. Recent experience with similar health threats: Places that have had a recent past adverse experience with a public health threat may be quicker to act in response to a new similar threat. For example, in response to COVID-19, Taiwan and Singapore, which were hit hard by SARS a decade earlier, acted very quickly and decisively, while countries like Canada and US, which had no major epidemics for over half a century, dithered.^105^
3. Ties between the government and industries (e.g., food or pharmaceutical industries): Agreements between for-profit industries and public health institutions can promote innovation (e.g., development of a new drug), but can also lead to public health decisions that favor these industries’ interests.^106^ It is often hard to identify such ties and the various ways they affect policy outcomes.
4. Agencies’ organizational structure: Public health agencies’ structure can favor one policy choice over another. For example, organizational structures that aim to limit one type of error (e.g., false positive) often leads to greater number of other types of errors (e.g., false negative).^107^
5. Social assumptions of racial differences: Beliefs about racial differences can affect public health choices and recommendations. For example, in the in the United States, the belief that African Americans have denser bones and thicker skin and muscles resulted in larger x-ray doses to make diagnostic pictures. This belief and recommendation had appeared in standard x-ray technology textbooks until the mid-1960s. Consequently, African Americans were getting increased radiation doses compared to other populations, which can have lasting negative health effects.^108^
6. Cognitive biases and constraints on information processing: Flawed human judgment and choice among policymakers can lead to poor policy decisions. A limited capacity to process information or systematic error in judgment can lead policymakers to make mistaken decisions.^109^
7. Political considerations: Policymakers seeking reelection often overreact to voters’ opinions for credit-claiming purposes or underreact to avoid the blame associated with a certain policy.^110^ For example, deliberate over and underreactions against COVID-19 in Israel reflected political considerations benefiting Prime Minister Netanyahu.^111^

We should also expect that combinations of two or more mechanisms sometimes lead to errors. For example, consider *agency organizational structure* and *ties between the government and industries* mechanisms: an agency’s organizational structure can permit and encourage collaboration with for-profit pharmaceutical companies. Such ties can lead to industry influence on agency officials and consequently to policies that favor its interest over the public’s. In such cases, a combination of the mechanisms can lead to public health errors. There are certainly more mechanisms like the above that could be at play and lead to errors, and the suggested definition of public health errors encourages researchers to pay attention to general mechanisms of this sort.

To explain why these mechanisms are general, consider, for example, how the *scientific uncertainty* mechanism can lead to different types of errors. Public health officials can take actions, relying on the best available data, that could not reasonably be predicted to be inefficient or cause harm. For example, in drug regulation, the precise safety and efficacy profile of any molecule is impossible to know, and therefore, rare adverse effects are possible.^112^ Such uncertainties can lead to the approval of a faulty drug (error of commission) or to the failure to discover and effectively respond to adverse effects for drugs already on the market (error of omission). In other words, scientific uncertainties can lead public health actors, doing what they could to choose the best actions, to take actions that cause direct harm (error of commission) or to not take sufficient action to address a new public health threat (error of omission). In both cases, the limits of scientific knowledge at a certain point in time can lead to non-culpable errors of commission or omission.

Scientific uncertainties can also lead to public health decisions that carry some degree of culpability. For example, I suggest culpability is present when public health actors, facing uncertainties, do not do what is needed to address these uncertainties (e.g., do not adequately assess potential health risks) and take actions that retrospectively prove to be in error. Similar to the examples above, this mechanism can lead to errors of commission or omission, but public health officials could have acted in a more advantageous way to address these uncertainties. In all cases, the *scientific uncertainty* mechanism can lead public health officials to make decisions whose consequences for public health were substantially worse than those of an alternative that could have been chosen instead, whether they are culpable or not, or by commission or omission.

As another example, consider the *ties between the government and industries* mechanism. This mechanism does not necessarily lead to culpable errors. For example, because the Canadian health regulator (Health Canada) relies on information provided by drug companies in the post-marketing stage (along with safety information collected from other sources), the agency is more susceptible to industry tactics to obscure evidence about how their products can harm patients.^113^ Consequently, in some cases, health officials, though in good faith doing what they can to detect new health risks associated with a drug, may fail to fully understand the potential risks of a drug due to industry efforts to hide this information. If this is the case, it would be debatable whether the error carries a degree of culpability or not, because culpability may be understood to require some negligence on the part of public health officials.^114^ However, as highlighted before, the focus of the proposed concept of public health errors is on the mechanisms, in general, that can cut across the four types of errors, rather than on categorizing them into subtypes.^115^

Once the error is identified and mechanisms are recognized, an investigation into whether the error carries a degree of culpability can follow. Focusing on general mechanisms that can lead to errors, rather than on culpability, is also beneficial for public health actors interested in preventing such errors. Developing strategies only to prevent culpable errors (for example, stricter oversight to prevent unjustified departures from established decision-making procedures), though important, overlook a wide range of non-culpable and often harmful errors. Once a mechanism (or mechanisms) is identified, efforts can be made to prevent the possibility of similar errors in the future.

## 5. Case Studies

In this section I assess two case studies: radiation treatment for benign conditions, and the opioid epidemic in the US and Canada. I illustrate the usefulness of the newly developed conception of public health errors in evaluating these cases and key implications learned from this analysis.

### 5.1 Radiation Treatment

Radiation treatment for benign illnesses (that is, not for treating cancer) was common worldwide between 1910 and 1960. The use of low-dose radiation was considered to be a safe and effective treatment for a variety of benign illnesses, such as cervical adenitis, hemangiomas of the head and neck, acne, tinea capitis (ringworm), birthmarks, infertility, pertussis, deafness, hypertrophy of the tonsils and adenoids, enlargement of the thymus gland (which was incorrectly believed to cause crib death), and more.^116^ In the early 1970s, when the treatment was no longer in use, epidemiological research confirmed that children and young adults treated with radiation for non-cancerous conditions showed an alarming tendency to develop brain tumors, thyroid cancer, and other ailments as adults.^117^

In this case a different decision — to treat benign conditions by means that did not involve low-dose radiation — clearly would have yielded better health outcomes (thus, an “error”). The radiation case study also connects to the first general mechanism leading to public health errors presented in the previous section: *scientific uncertainty*. The decision to use ionizing radiation in hospitals and public health campaigns worldwide was based on what was known at the time — low-dose radiation was considered safe and effective. The late health effects, not understood at the time, were discovered 10 to 30 years after millions of children and young adults had received the treatment, and when radiation was no longer in use to treat benign conditions. In other words, what was considered to be a safe and effective treatment at the time by the scientific medical community turned out to have deadly long-term health effects. This case study demonstrates that the impacts of public health interventions are sometimes difficult to predict because of the potential for rare long-term health effects. Therefore, when assessing current cases of adverse effects of public health intervention (e.g., the ongoing COVID-19 pandemic), it is critical to acknowledge and consider the temporal dimension.

Furthermore, I have shown elsewhere^118^ that the discovery of similar adverse effects of radiation treatment (e.g., thyroid cancer, brain tumors) led to broken trust and suspicion toward the medical establishment in Israel, yet triggered no such hostility or broken trust regarding US health authorities. These findings suggest that distrust is not tied to culpability. Both the Israeli and American patients experienced similar severe late health effects, while only in Israel did the discovery led to harsh feelings toward and mistrust of the national health institutions. The comparison challenges the belief that only culpable errors would lead to mistrust (because some negligence was associated with the action). Therefore, one implication of analyzing this case is that if we want to better understand the social effect of errors on the public (e.g., mistrust), it is important to focus on both culpable and non-culpable errors. It is more likely that the negative outcome — regardless of culpability — together with structural factors (such as social inequalities and feelings of exclusion from the dominant elite) lead to mistrust.^119^

The radiation case study illustrates that even if the original error was not culpable, it is important to respond to it promptly and transparently. Promptly, because early detection of the adverse effects (e.g., the early detection of thyroid cancer) can save lives. Transparently, because it promotes trust in the medical establishment and because the error may be perceived as malicious if its harms were borne disproportionately by socially disadvantaged groups (as happened in the Israeli case). That being said, transparency alone might not be sufficient for marginalized populations experiencing the harms. These populations often have low trust in the government because of a history of racism, discrimination, or unethical medical experiments conducted on their members.^120^ Thus, specific strategies to communicate with marginalized populations experiencing harm — taking into consideration past and current mistrust toward the government — are often needed.^121^ The paternalistic and non-transparent approach by the Ministry of Health in Israel toward disadvantaged groups experiencing the adverse effects of radiation treatment^122^ and the hostility it created toward health authorities^123^ demonstrate the negative consequences that can result when these factors are not considered. In sum, even if the error was not culpable because it was based on the best the scientific knowledge at the time, studying the case can help us learn how to better respond to similar errors.

### 5.2 Opioid Epidemic

The opioid epidemic is one of the worst public health crises in the history of the United States and Canada, with more than 67,700 opioid-related deaths in the United States in 2020^124^ and 6,214 in Canada in 2020.^125^ Here I focus on the FDA’s erroneous decision to include false information on the OxyContin labeling, and Health Canada’s (HC) delayed and inadequate response to evidence of addiction and misuse associated with OxyContin. These two errors, I show, have contributed to the opioid epidemic in North America.

The FDA’s approval of OxyContin^126^ in 1995 and Purdue Pharma’s aggressive marketing campaign that followed are recognized as major factors leading to the overprescription of opioids in the United States.^127^ Excessive prescription of opioids is associated with high rates of overdose deaths and considered to be a root cause of the current crisis.^128^ When the FDA approved OxyContin in 1995, the drug’s label stated that the drug had low misuse and addiction liability and would be effective for moderate to severe pain for the long-term. In reality, OxyContin is a highly addictive opioid that can be easily misused,^129^ with limited effectiveness for relieving pain over the long-term.^130^

In fact, there was no evidence that the risk of addiction was low or that it carried less misuse potential than other oxycodone opioids.^131^ Furthermore, the misleading and confusing sentence, “Delayed absorption, as provided by OxyContin tablets, is believed to reduce the abuse liability of a drug,” does not meet the FDA’s criterion of providing informative and accurate information, as opposed to implied claims and suggestions if there is no evidence of effectiveness and safety.^132^ Furthermore, the misuse liability of OxyContin was, in fact, greater because of the large quantity of pure oxycodone in each OxyContin pill and the ease of accessing it all at once by crushing the pills.^133^ In addition, little evidence supported the claim that OxyContin was good for moderate pain for long-term use (“more than a few days”).^134^

Purdue Pharma, permitted by law to use FDA-approved information in promotional practices, used this erroneous information as its principal selling point, which was key in promoting the drug.^135^ This consequently contributed to the excessive prescription of a potent and highly addictive drug and to the high rates of overdose deaths that followed.^136^

FDA officials later acknowledged that the initial wording of OxyContin’s label was “unfortunate.”^137^ One official noted, “[w]e began to become actually aware of how inaccurate the original label was and how it had probably contributed to the problem.”^138^ The problem the official referred to was the deaths of healthy people from OxyContin use and misuse.^139^ On the “60 Minutes” program, David Kessler, FDA commissioner from 1990 to 1997, noted that “there are no studies on the safety or efficacy of opioids for long-term use.” ^140^

The mistaken information on the label was based on little to no evidence. Even if the FDA believed that the controlled-release formulation would have minimized the risk of misuse and addiction, it should have based its decision regarding the label information on evidence supporting these claims. The FDA committed a preventable error that helped initiate the opioid prescription epidemic. “No doubt it was a mistake,” Kessler noted, referring to the belief that opioids are safe and effective for chronic pain. “It was certainly one of the worst medical mistakes, a major mistake.”^141^

This evidence suggests that the FDA’s actions carry some degree of culpability. The FDA could have acted in a more advantageous way, first by carefully reviewing the evidence before deciding on the label information, and second by making claims based on data, especially considering the potency of OxyContin. This is an *error* because this decision clearly caused significant harm compared to a decision not to include this misleading information on the drug’s label.

A less known, but equally significant, error was made by HC, the Canadian health regulator. I have shown elsewhere that the misleading information that appeared in the OxyContin product monograph in Canada was used by Purdue Pharma in its promotional practices to mislead health professionals in Canada to increase prescription.^142^ I have focused on HC’s delayed response (updating the monograph) to new evidence of risk, suggesting that despite strong evidence of addiction and misuse of the drug, it took HC more than 10 years to update the drug’s product monograph, and that the revision was inadequate, which may have contributed to the overprescription epidemic of opioids in Canada and to opioid-related deaths. I have previously argued that not taking a stronger action (e.g., acting faster and deleting misleading information that appeared in the monograph) was wrong and failed to prevent unnecessary harm, including deaths.^143^

HC’s omission involves some degree of culpability because, despite strong evidence, the agency did not take the necessary actions to prevent harm, believing that OxyContin misuse problems were limited to the US. Most notably, HC did not follow the FDA’s decision in 2001 to delete misleading claims that appeared on the OxyContin label and to add a Black Box Warning to the drug packaging. It is an *error* because a different action — promptly deleting misleading claims and alerting physicians of the danger — could have clearly prevented significant harm.

The opioid case study connects to the *ties between the government and industries* mechanism presented in section 4. It raises the possibility that the FDA and HC ignored evidence regarding the risks of opioids because of close ties between the agencies and pharmaceutical companies. In the US, collaboration between the FDA officials responsible for approving new drugs and drug manufacturers may have led to the FDA’s decision to include inaccurate, false, and confusing information on the OxyContin label, based on little or no evidence.^144^ Mislabeling the drug also raises the possibility that the FDA was “captured” by drug companies. In this context, capture can be defined as “the result or process by which regulation, in laws or application, is consistently or repeatedly directed away from the public interest and toward the interest of the regulated industry, by the intent and action of the industry itself.”^145^ In addition, the approval of OxyContin may have been aided by a “revolving doors” mechanism, a situation in which officials leave the FDA for lucrative jobs in the pharmaceutical industry.^146^ Dr. Curtis Wright, the supervisor of the FDA team that examined Purdue’s OxyContin application, later left the FDA to work for Purdue.^147^

Similarly, HC’s omission might be due to close relationship between the industry and the regulator — as HC saw the pharmaceutical industry as major partner in drug regulation.^148^ Such ties between the HC and drug companies may explain HC’s poor response to the adverse effects associated with OxyContin. Collaboration between HC and the industry, as well as a history of “regulation through cooperation,”^149^ in which HC relies on industry for self-regulation, could have prompted the regulator to make decisions that favored the industry’s interest over the public’s. As I have shown elsewhere,^150^ HC ignored evidence about the drug’s adverse effects; did not following its own post-marketing standard (most notably, not following the FDA’s actions); delayed its decision to correct misleading information that appeared in the monograph; and inadequately revised the monograph (only slightly revising the misleading sections).

In sum, ties between the government and industries may have led to an error of commission in the United States and of omission in Canada, and both these errors involve some degree of culpability. Namely, officials ignored evidence about the risks associated with the opioid OxyContin in the pre- (US) and post-market (Canada) stages. In both cases, the two regulators could have better reviewed the data and taken different actions that would have better protected the public’s health.

A key lesson from the opioid case studies is that systemic reform is needed to moderate the problematic relationships between pharmaceutical companies and health regulators to prevent drug companies from adversely influencing the medical community and population health. I support existing proposals that call for a reform in the regulation of pharmaceutical companies in North America. For example, I endorse calls to give drug regulators more independence and power to act in the pre- and post-approval of pharmaceuticals; to restrict membership on regulatory advisory committees to experts without financial ties to drug companies; to end all industry funding and financial relationships with regulatory and health technology assessment agencies; to establish new public agencies to fund drug development and clinical trials; and to insist that the promotion of drugs be more strictly overseen by regulatory authorities.^151^ While most discussion on the opioid crisis has focused on Purdue’s promotional tactics of OxyContin,^152^ similar tactics are common across the pharmaceutical industry.^153^ Thus, merely imposing legal penalties on Purdue and FDA officials who approved OxyContin is unlikely to prevent future catastrophes of this kind. This is why it is important to consider public health errors in relation to systemic mechanisms that are likely to persist even after blameworthy actors, such as Purdue Pharma and the FDA officials who approved OxyContin, have been held accountable or left the scene. This further reinforces the claim that it is better not to overemphasize culpability in connection with public health errors.

Furthermore, the radiation and opioid examples illustrate that whether an error is culpable or not, it may lead to a similar negative outcome, both in terms of negative health outcomes and distrust of health officials. This gives further support to my decision not to rely on culpability as a necessary condition for a public health error to occur. Focusing merely on culpability would mean the radiation case would not be categorized as a public health error (despite similar outcomes). It also illustrates the usefulness of focusing on general mechanisms that can lead to public health errors, whether culpable or not, by omission or commission.

While it is widely recognized that public health interventions, like medical interventions, can err, the concept of public health error has received surprisingly little attention, and disagreements exist about how the concept should be defined.

## 6. Conclusions and Implications

While it is widely recognized that public health interventions, like medical interventions, can err, the concept of public health error has received surprisingly little attention, and disagreements exist about how the concept should be defined. In this paper, I propose that “public health error” be defined as an act of commission or omission, culpable or not, by public health officials whose consequences for population health were substantially worse than those of an alternative that could have been chosen instead. Based on the above conception, I have suggested that (1) the definition of public health errors includes interventions that directly caused harm to population health and were worse than doing nothing at all; (2) the definition also includes failure to take action when measures were needed to protect the health of the population; (3) public health measures that do some good (i.e., improve population health), but which are still significantly inadequate in comparison to alternatives, are public health errors; (4) interventions that were only slightly suboptimal would not be counted as public health errors because they are not substantially worse (i.e., they don’t entail a significant degree of error); and (5) grey area cases, in which judgment about which intervention is best depends on debatable ethical premises, would not be counted as public health errors.

The newly developed conception’s main advantages are that it better fits the work of public health (focusing on health outcomes rather than on political or other goals) and it avoids relying on culpability as a necessary condition for public health error to occur. From a practical perspective, not making attribution of blame part of the definition makes it easier to apply in practice and better corresponds with the common usage of the term. From a theoretical perspective, it is better to have a public health error concept that does not require culpability, because culpable and non-culpable errors can have similar causes and effects. The proposed concept thus permits the consideration of general mechanisms leading to public health errors that can be relevant for any of the four combinations (omission, commission, culpable, non-culpable) and cut across these types.

I have also demonstrated that the proposed concept of public health errors is a fruitful lens for analyzing very different case studies involving harms of public health interventions. I have shown that public health campaigns to eradicate benign conditions using low-dose radiation (a non-culpable error of commission), the opioid epidemic in the US (a culpable error of commission) and the opioid epidemic in Canada (a culpable error of omission) represent three subtypes of public health error. These cases illustrate that whether an error is culpable or not, it may lead to similar negative outcomes and that mistrust is not necessarily tied to culpability. Based on the analysis of the radiation case study, I have suggested that promptly responding to errors, transparently sharing information with the public, and developing strategies to communicate with marginalized populations are critical even if the error was not culpable. Based on the opioid cases study, I have recommended a reform of the pharmaceutical systems in North America that regulates the problematic relationship between the industry and health regulators such as HC and the FDA.

The implications of this study may help us understand current polices to contain the COVID-19 global pandemic. Governments’ responses to the novel coronavirus raise questions regarding the appropriate public health interventions to contain the virus and mitigate the harm.^154^ The discussions around COVID-19 also raise the question whether national health authorities committed public health errors in their public health responses to the virus (e.g., delayed travel restrictions, lockdowns, school closures). The concept presented here can potentially assist scholars and public health actors who are interested assessing these actions, clarifying some of these questions and helping to prevent similar errors. Further research to identify new mechanisms leading to public health errors, using the newly developed conception, will enhance our understanding of their occurrence.

## References

[1] D. Holtgrave, “12 Ways the Trump Administration Botched America’s Response to Covid-19,” *CNN*, October 29, 2020, *available at* <https://edition.cnn.com/2020/10/29/opinions/ways-trump-botched-covid-response-holtgrave/index.html> (last visited October 1, 2021).

[2] D. Sokol , “‘First Do No Harm’ Revisited,” BMJ 347 (2013): f6426. 24163087 10.1136/bmj.f6426

[3] B. M. Dickens , “Medical Errors: Legal and Ethical Responses,” International Journal of Gynecology and Obstetrics 81, no. 1 (2003): 109–114; D. Holtgrave, “Public Health Errors: Costing Lives, Millions at a Time,” *Journal of Public Health Management & Practice* 16, no. 3 (2010): 211–215; F. Rosner et al., “Disclosure and Prevention of Medical Errors,” *Archives of Internal Medicine* 160, no. 14 (2000): 2089–2092; M. A. Makary and M. Daniel “Medical Error — The Third Leading Cause of Death in the US,” *BMJ* 353 (2016): i2139.12676409

[4] Cited in Holtgrave, *supra* note 3, at 214.

[5] See Dickens, *supra* note 3; T.H. Gallagher et al., “Patients’ and Physicians’ Attitudes Regarding the Disclosure of Medical Errors,” Journal of the American Medical Association 289, no. 8 (2003): 1001–1007; K. Mazor et al., “Communicating with Patients about Medical Errors,” *Archives of Internal Medicine* 164, no. 15 (2004): 1690–1697; Rosner et al., *supra note* 3.12597752

[6] See J.E. Childress et al., “Public Health Ethics: Mapping the Terrain,” Journal of Law, Medicine & Ethics 30, no. 2 (1988): 170–178; Holtgrave, *supra note* 3; N. Kass, “An Ethics Framework for Public Health,” *American Journal of Public Health* 91, no. 11 (2001): 1776–1782; K. De Ville and L. F. Novick, “Toward a Taxonomy of Public Health Error,” *Journal of Public Health Management and Practice* 16, no. 3 (2010): 216–220.

[7] See D. Carpenter and and M. Ting , “Regulatory Errors with Endogenous Agendas,” American Journal of Political Science 51, no. 4 (2007): 835–852; L. C. F. Heimann, *Acceptable Risks: Politics, Policy, and Risky Technologies* (Ann Arbor: University of Michigan Press, 1997).

[8] For example, see C. Bonell , et al., “‘Dark Logic’: Theorising the Harmful Consequences of Public Health Interventions,” Journal of Epidemiology and Community Health 69, no. 1 (2015): 95–98; T. Lorenc and K. Oliver, “Adverse Effects of Public Health Interventions: A Conceptual Framework,” *Journal of Epidemiology and Community Health* 68, no. 3 (2014): 288–290; S. Macintyre, and M. Petticrew, “Good Intentions and Received Wisdom are Not Enough,” *Journal of Epidemiology and Community Health* 54 (2000): 802-803.25403381

[9] See Holtgrave, *supra* note 3; De Ville and Novick, *supra* note 6.

[10] With the exception of Holtgrave *supra* note 3, studies do not provide a definition of public health errors.

[11] D. Carpenter and and M. Ting , “Essay: The Political Logic of Regulatory Error,” Nature Reviews Drug Discovery 4, no. 10 (2005): 819–823; Holtgrave, *supra* note 3; De Ville and Novick, *supra* note 6. 16184084 10.1038/nrd1850

[12] See M. Bovens and P. Hart , Understanding Policy Fiascoes (New Brunswick: Transaction Books, 1996); M. Howlett, “Policy Analytical Capacity and Evidence‐based Policy‐Making: Lessons from Canada,” C*anadian Public Administration* 52, no. 2 (2009): 153–175; B. Hudson et al., “Policy Failure and the Policy-Implementation Gap: Can Policy Support Programs Help?” *Policy Design and Practice* 2, no. 1 (2019): 1–14; A. McConnell, *Understanding Policy Success: Rethinking Public Policy* (New York: Macmillan International Higher Education, 2010); J. Walsh, “Policy Failure and Policy Change: British Security Policy after the Cold War,” *Comparative Political Studies* 39, no. 4 (2006): 490–518.

[13] Heimann, *supra* note 7; L. C. F. Heimann , “Understanding the Challenger Disaster: Organizational Structure and the Design of Reliable Systems,”*American* Political Science Review 87, no. 2 (1993): 421–435; Carpenter and Ting, *supra* note 7.

[14] Institute of Medicine, The Future of Public Health (Washington: The National Academies Press, 1988). 25032306

[15] J. Dupré , “Natural Kinds and Biological Taxa,” The Philosophical Review 90, no. 1 (1981): 66–90; P. Kitcher, “Species,” *Philosophy of Science* 51, no. 2 (1984): 308–333.

[16] I. Bavli and D. Steel , “On Community Epistemic Capacity,” Social Epistemology Review and Reply Collective 4, no. 12 (2015): 34–38.

[17] See Bonell et al., *supra* note 8; Lorenc and Oliver, *supra* note 8; Macintyre and Petticrew, *supra* note 8.

[18] Holtgrave, *supra* note 3; De Ville and Novick, *supra* note 6.

[19] See Carpenter and Ting, *supra* note 7; Carpenter and Ting, *supra* note 11; Heimann, *supra* note 7; M. Howlett , “The Lessons of Failure: Learning and Blame Avoidance in Public Policy-Making,” International Political Science Review 33, no. 5 (2012): 539–55; A. McConnell, “What Is Policy Failure? A Primer to Help Navigate the Maze,” *Public Policy and Administration* 30, no. 3–4 (2015): 221–242; J. Rachlinski and J., and C. R. Farina, “Cognitive Psychology and Optimal Government Design,” *Cornell Law Review* 87 (2001): 549–615.

[20] Institute of Medicine, *supra* note 14.

[21] R. Upshur , “Principles for the Justification of Public Health Intervention,” Canadian Journal of Public Health 93, no. 2 (2002): 101–103. 11968179 10.1007/BF03404547 PMC6979585

[22] Institute of Medicine, The Future of the Public’s Health in the 21st Century (Washington: The National Academies Press, 2003).

[23] Institute of Medicine, *supra note* 14; Kass, *supra* note 3.

[24] Macintyre and Petticrew, *supra* note 8.

[25] Lorence and Oliver, *supra* note 8.

[26] Bonell et al., *supra* note 8.

[27] M. Wiggins et al., “Health Outcomes of Youth Development Programme in England: Prospective Matched Comparison Study,” BMJ 339 (2009): b2534. 19584408 10.1136/bmj.b2534 PMC2714682

[28] Bonell et al., *supra* note 8.

[29] I am treating the approval and rejections of drugs as public health decisions.

[30] For discussions on the the painkiller Vioxx, see section 2.2; for discussions on the opioid epidemic, see section 5.2.

[31] Cited in Holtgrave, *supra* note 3.

[32] Holtgrave, *supra* note 3.

[33] *Id*.

[34] De Ville and Novick, *supra* note 6.

[35] *Id*.

[36] For example, in agencies such as the U.S. Food and Drug Administration (FDA), National Aeronautics and Space Administration (NASA), and the Environmental Protection Agency (EPA). See Heimann, *supra* note 7.

[37] See Carpenter and Ting, *supra* note 7; Carpenter and Ting, *supra* note 11.

[38] Heimann, *supra* note 7.

[39] Drawing on the statistical notion of type 1 (false positive) and type 2 (false negative) errors, Heimann argued that agencies will choose to divide their resources and efforts between the two types of errors in a way that reduces the chance that the more costly error will occur, assuming that each type of error is associated with a different set of costs. The organizational structure that aims to limit just one type of error often leads to a greater number of other types of error. Drawing on two case studies, the Challenger disaster (type 1 error) and the delayed approval of HIV/AIDS medication (type 2 error), he illustrated how attempts to balance between the two types of errors (among other factors) can potentially explain these errors. The idea that there is a balance between type 1 and 2 errors, or over- and under-regulation, was discussed by J. Stegenga , “Drug Regulation and the Inductive Risk Calculus,” in K. C. Elliott and T. Richards , eds., Exploring Inductive Risk: Case Studies of Values in Science (New York: Oxford University Press, 2017): 17–36; and I. Bavli and D. Steel, “Inductive Risk and OxyContin: The Ethics of Evidence and Post-Market Surveillance of Pharmaceuticals in Canada,” *Public Health Ethics* 13, no. 3 (2020): 300–313.

[40] Heimann, *supra* note 7; Heimann, *supra* note 13.

[41] Carpenter and Ting, *supra* note 7; Carpenter and Ting, *supra* note 11.

[42] They investigated two case studies: the painkiller Vioxx, which was taken off shelves because it was associated with high rates of heart attacks and deaths (type 1 error), and beta-blockers, a safe and effective hypertension drug that the FDA delayed approving (See Carpenter and Ting, *supra* note 11).

[43] Carpenter and Ting, *supra* note 11.

[44] Holtgrave, *supra* note 3.

[45] Adverse drug events (also called “side effects”) are defined as: “any undesirable experience associated with the use of a medicine in a patient … can range from mild to severe. Serious adverse events are those that can cause disability, are life-threatening, result in hospitalization or death, or are birth defects.” See: Food and Drug Adminstration, “A Guide to Drug Safety Terms at FDA,” *Consumer Health Information* (2012), *available at* <https://www.fda.gov/consumers> (last visited October 4, 2021).

[46] Carpenter and Ting, *supra* note 11. They also highlight the difficulties in assessing FDA errors. For example when discussing the Vioxx and beta-blockers case studies, they noted that these are not necessarily examples of regulatory errors because of their incapability to assess the many uncertainties around the specific features of both cases.

[47] Carpenter and Ting, *supra* note 11.

[48] See Food and Drug Adminstration, *supra* note 45; H. Nachlis , “Pockets of Weakness in Strong Institutions: Post-Marketing Regulation, Psychopharmaceutical Drugs, and Medical Autonomy, 1938–1982,” Studies in American Political Development 32, no. 2 (2018): 257–291.

[49] Carpenter and Ting, *supra* note 11.

[50] Carpenter and Ting, *supra* note 7.

[51] To measure errors, they look at two sets of measures: (1) any significant label revisions made by the FDA (measured by the number of lines of text describing major labeling revisions); and (2) action by foreign regulators (in highly developed countries that are independent from the FDA): the removal from the market of the same drug that was approved by the FDA, as well as markets where the drug was never entered.

[52] Carpenter and Ting, *supra* note 7.

[53] Carpenter and Ting, *supra* note 11.

[54] Carpenter and Ting, *supra* note 7.

[55] For example, J. Buchanan , “Drug Policy under New Labour 1997-2010: Prolonging the War on Drugs,” Probation Journal 57, no. 3 (2010): 250–262; D. P. Francis, “Deadly AIDS Policy Failure by the Highest Levels of the US Government: A Personal Look Back 30 Years Later for Lessons to Respond Better to Future Epidemics,” *Journal of Public Health Policy* 33, no. 3 (2012): 290–300; J. Hammer et al, “Understanding Government Failure in Public Health Services,” *Economic and Politically Weekly* 42, no. 40 (2007): 4049–4057. 10.1057/jphp.2012.14 22895498

[56] See D. K. Gupta , Analyzing Public Policy: Concepts, Tools, and Techniques (Washington: CQ Press, 2001); B. Hogwood and L. A. Gunn, *Policy Analysis for the Real World* (New York: Oxford University Press, 1984).

[57] See M. Fotaki , “Why Do Public Policies Fail so Often? Exploring Health Policy-Making as an Imaginary and Symbolic Construction,” Organization 17, no. 6 (2010): 703–720; B. Mueller, “Why Public Policies Fail: Policymaking under Complexity,” *EconomiA* 21, no. 2 (2020): 311–323; D. Wallinga et al., “Antimicrobial Resistance and Biological Governance: Explanations for Policy Failure,” *Public Health* 129, no. 10 (2015): 1314–1325. 10.1016/j.puhe.2015.08.012 26454427

[58] For example, Bovens and Hart, *supra* note 12; Howlett, *supra* note 19; McConnell, *supra* note 19.

[59] Howlett , *supra* note 19.

[60] See, for example, C. Hood , The Blame Game: Spin, Bureaucracy, and Self-Preservation in Government (Princeton : Princeton University Press, 2011); J. Kingdon, *Agendas, Alternatives, and Public Policies* (New York: Longman, 1995); Walsh, *supra* note 12.

[61] The *process* dimension refers to governments’ failure during the policymaking process to produce policy decisions, i.e., to successfully proceed from policy ideas and complete the policy process (for example, governments can fail to gain authoritative approval of a particular policy initiative). The *program* refers to the failure to meet original pragmatic goals or technical goals (for example, failure to achieve desired outcomes, implementing the program as intended, satisfying criteria highly valued in policy domain or attracting support for the program). The *politics* dimension (policy failure as a political issue) refers to the political consequences of policy outcomes (e.g., failing to gain the political support after implementation or long-term damage to an agency’s reputation). See: McConnell, *supra* note 20; McConnell, *supra* note 12.

[62] For discussion of these topics, see Bovens and Hart, *supra* note 12; Howlett, *supra* note 12; Howlett, *supra* note 19, Hudson et al., *supra* note 12; McConnell, *supra* note 12, McConnell, *supra* note 19; Walsh, *supra* note 12.

[63] McConnell, *supra* note 19.

[64] *Id*.

[65] *Id*.

[66] Holtgrave*, supra* note 3.

[67] Carpenter and Ting, *supra* note 11.

[68] See Bovens and Hart, *supra* note 12; Hood, *supra note* 60; Kingdon, *supra* note 60; Walsh, *supra* note 12.

[69] Bovens and Hart, *supra* note 12.

[70] Howlett, *supra* note 19.

[71] Rachlinski and Farina, *supra* note 19.

[72] See Bonell et al., *supra* note 8, Lorence and Oliver, *supra* note 8; Macintyre and Petticrew, *supra* note 8; De Ville and Novick, *supra* note 6.

[73] See Bonell et al., *supra* note 8, Lorence and Oliver, *supra* note 8; Macintyre and Petticrew, *supra* note 8.

[74] Carpenter and Ting, *supra* note 7, Carpenter and Ting, *supra* note 11; Holtgrave, *supra* note 3; De Ville and Novick, *supra* note 6.

[75] Howlett, *supra* note 19; McConnell, *supra* note 19.

[76] Bonell et al., *supra* note 8.

[77] See Holtgrave, *supra* note 3.

[78] S. Anand . “The Concern for Equity in Health,” in Public Health, Ethics, and Equity, eds. Anand et al. (New York: Oxford University Press, 2006): 15–20.

[79] Childress et al., *supra* note 6.

[80] I. Bavli et al., “Harms of Public Health Interventions against Covid-19 Must Not Be Ignored,” BMJ 371: m4074. 10.1136/bmj.m4074 33139247

[81] See I. Bavli , “Industry Influence and Health Canada’s Responsibility: Lessons from the Opioid Epidemic in Canada,” Addiction 115, no. 9 (2000): 1605–1606; Bavli and Steel, *supra* note 39. 10.1111/add.14929 31845436

[82] Thalidomide was approved in Canada in 1961 (it was also previously approved in other European countries, but not in the United States). The drug had been prescribed to pregnant women to relieve pregnancy nausea. It caused irreversible fetal damage, resulting in thousands of children being born with severe congenital malformations. See Thalidomide Victim Association of Canada, *available at* <https://thalidomide.ca/en/> (last visited October 5, 2021).

[83] For more discussions about the failure to act against the harmful effects of tobacco in the United States, see A. M. Brandt , “Inventing Conflicts of Interest: A History of Tobacco Industry Tactics,” American Journal of Public Health 102, no. 1 (2012): 63–71; A. M. Brandt, *The Cigarette Century: The Rise, Fall, and Deadly Persistence of the Product That Defined America* (New York: Basic Books, 2007); R. Proctor, *Golden Holocaust: Origins of the Cigarette Catastrophe and the Case for Abolition* (Berkeley and Los Angeles: University of California Press, 2011). 22095331 10.2105/AJPH.2011.300292 PMC3490543

[84] For more discussions on how to evaluate harms against benefit, see J. Dumit , Drugs For Life: How Pharmaceutical Companies Define Our Health (Durham and London: Duke University Press, 2012) on the notion that we should value new drugs relative to their side effects; and J. Avorn and A. Kesselheim, “Regulatory Decision-Making on COVID-19 Vaccines during a Public Health Emergency,” *JAMA* 324, no. 13 (2020): 1284–1285, on the challenges of the attempts to develop a safe vaccine for SARS-CoV-2. 10.1001/jama.2020.17101 32870268

[85] De Ville and Novick, *supra* note 6. It also includes instances of industry influence that can prompt public health agencies to make a poor decision that favors the company’s interest. See D. Carpenter and D. Moss , Preventing Capture: Special Interest Influence in Regulation, and How to Limit It (New York: Cambridge University Press, 2014); T. Makkai and J. Braithwaite, “In and Out of Revolving Door: Making Sense of Regulatory Capture,” *Journal of Public Policy* 12, no. 1 (1992): 61–78; P. J. Quirk, “Food and Drug Administration,” in *The Politics of Regulation*, ed. J. Q. Wilson (New York: Basic Books, 1980): 191–235; G. J. Stigler, “The Theory of Economic Regulation,” *The Bell Journal of Economics and Management Science* 2, no. 1 (1971): 3–21.

[86] See Thalidomide Victim Association of Canada, *supra* note 82. In fact, there was no evidence at the time of approval that the drug was safe to use. The FDA, on the other hand, did better research assessing the risk: it ended up not approving the drug and preventing the error (ironically, it was a Canadian — Frances Oldham Kelsey, who joined the FDA shortly before Canada approved the drug — who blocked FDA approval of Thalidomide in the United States).

[87] Note that the tobacco industry played a pivotal role in obscuring the evidence and manipulating scientific evidence. For discussions on this topics, see A. M. Brandt , “Inventing Conflicts of Interest: A History of Tobacco Industry Tactics,” American Journal of Public Health 102, no. 1 (2012): 63–71; D. Michaels, *The Triumph of Doubt: Dark Money and the Science of Deception*, (New York: Oxford University Press, 2020); N. Oreskes and E. M. Conway, *Merchants of Doubt: How a Handful of Scientists Obscured the Truth on Issues from Tobacco Smoke to Global Warming* (New York: Bloomsbury Press, 2011).22095331

[88] See G. Markowitz and D. Rosner , Deceit and Denial: The Deadly Politics of Industrial Pollution (Berkeley and Los Angeles: University of California Press, 2002); D. Rosner and G. Markowitz, “Building the World That Kills US: The Politics of Lead, Science, and Polluted Homes, 1970 to 2000,” *Journal of Urban History* 42, no. 2 (2016): 323–345.

[89] See S. Masten et al., “Flint Water Crisis: What Happened and Why?” American Water Works Association 108, no. 12 (2016): 22–34. 10.5942/jawwa.2016.108.0195 PMC5353852 28316336

[90] De Ville and Novick, *supra* note 6.

[91] Upshur, *supra* note 21; Institute of Medicine, *supra* note 14.

[92] See Bovens and Hart, *supra* note 12; Howlett, *supra* note 19; Howlett, *supra* note 12; Hudson et al., *supra note* 12; McConnell, *supra* note 19; McConnell, *supra* note 12; Walsh, *supra* note 12.

[93] For example, McConnell, *supra* note 19.

[94] For example, Howlett, *supra* note 19; McConnell, *supra* note 19; McConnell, *supra* note 12.

[95] McConnell, *supra* note 19; McConnell, *supra* note 12.

[96] Howlett, *supra* note 19; Howlett, *supra* note 12; Hudson et al., *supra* note 12.

[97] National Cancer Institute, *National Cancer Program, Special Communication: Irradiation-Related-Thyroid Cancer*, DHEW Publication No. [NIH] 77-1206 (July 13, 1977).

[98] N. Karako Eyal, “The Ringworm Case and the Lost Opportunities for the Construction of a Collective Healing Process,” *The International Journal of Conflict Engagement and Resolution* 5, no. 1-2 (2017): 25–51.

[99] See I. Bavli and S. Shvarts , “Michael Reese Hospital and the Campaign to Warn the US Public of the Long-Term Health Effects of Ionizing Radiation, 1973–1977,” American Journal of Public Health 109, no. 3 (2019): 398–405; S. Shvarts et al., “The Tinea Capitis Campaign in Serbia in the 1950s,” *The Lancet Infectious Diseases* 10, no. 8 (2010): 571–576. 30726139 10.2105/AJPH.2018.304763 PMC6366520

[100] Carpenter and Ting, *supra* note 11.

[101] *Id*. They also conditioned the concept of “error” by arguing that in addition to culpability, if the regulator appropriately acts to detect such adverse effects after approval and promptly responds to it (e.g., removing a drug from the market), it can be argued that no error was made. However, responses to mistakes are part of the post-marketing stage of pharmaceutical regulation and is irrelevant for the question of whether an error was made.

[102] See Z. Brzović, 2018. “Natural Kinds.” *The Internet Encyclopedia of Philosophy* (2018), *available at <* https://iep.utm.edu/nat-kind/> (last visited July 26, 2023).

[103] Dupré, *supra* note 16; Kitcher, *supra* note 16.

[104] McConnell’s (*supra* note 19) notion that a policy may succeed in the short-term but fail in the long-term is also applicable to public health errors. However, while McConnell refers also to long-term political failures or successes, I refer only to the effect of a public health choice on the public’s health.

[105] E. Han et al., “The Resilience of Taiwan’s Health System to Address the COVID-19 Pandemic,” EClinicalMedicine 24 (2000): 10437. 10.1016/j.eclinm.2020.100437 PMC7320260 32766544

[106] J. Marks , The Perils of Partnership: Industry Influence, Institutional Integrity, and Public Health (New York, NY: Oxford University Press, 2019).

[107] Heimann, *supra* note 7.

[108] I. Bavli and DS Jones , “Race Correction and the X-ray Machine — The Controversy over Increased Radiation Doses for Black Americans in 1968,” New England Journal of Medicine 387, no. 10 (2022): 947–952. 36069880 10.1056/NEJMms2206281

[109] Rachlinski and Farina, *supra* note 19; A. Tversky and D. Kahneman , “Availability: A Heuristic for Judging Frequency and Probability,” Cognitive Psychology 5, no. 2 (1973): 207–232.

[110] “Policy overreaction” refers to policies whose net cost outweighs the benefits; “policy underreaction” is a policy whose actual net utility is smaller than to the status-quo (a counterfactual net-utility). See M. Maor , “Policy Overreaction,” Journal of Public Policy 32, no. 3 (2021): 231–259; M. Maor, “Policy Persistence, Risk Estimation and Policy Underreaction,” *Policy Sciences* 47, no. 4 (2014): 425–443; M. Maor, “Deliberate Disproportionate Policy Response: Towards a Conceptual Turn,” *Journal of Public Policy* 41, no. 1 (2021): 185–208.

[111] M. Maor et al., “When COVID-19, Constitutional Crisis, and Political Deadlock Meet: The Israeli Case from a Disproportionate Policy Perspective,” Policy and Society 39, no. 3 (2020): 442–457. 35039730 10.1080/14494035.2020.1783792 PMC8754702

[112] Carpenter and Ting, *supra* note 11.

[113] Bavli and Steel, *supra* note 39; J. Lexchin , Private Profits versus Public Policy: The Pharmaceutical Industry and the Canadian State (Toronto: Toronto University Press, 2016). 10.12927/hcq.2017.25145 28550695

[114] Holtgrave, *supra* note 3.

[115] Note that ties between industry and the government would not necessarily lead to errors, as the successful collaboration between the Israeli government and Pfizer during the pandemic shows.

[116] See Bavli and Shvarts, *supra* note 99; National Cancer Institute, *supra* note 97; Shvarts et al., *supra* note 99.

[117] For discussions on the long-term health effects of ionizing radiation, see L. DeGroot , Radiation-Associated Thyroid Carcinoma. (New York: Grune & Stratton, 1977); B. Modan et al., “Radiation-Induced Head and Neck Tumours,” *The Lancet Infectious Diseases* 303, no. 7852 (1974): 277–79; E. Ron et al., “Tumors of the Brain and Nervous System after Radiotherapy in Childhood.” *NEJM* 319, no. 16 (1988): 1033–1039; B. Modan et al., “Increased Risk of Breast Cancer After Low-Dose Irradiation,” *The Lancet* 333, no. 8639 (1989): 629–631.

[118] Bavli and Steel, *supra* note 16.

[119] We suggest that important factors for understanding mistrust in health authorities include 1) which population is at risk (in Israel, the lower socioeconomic level and, in the US, the mostly white middle–upper class) and 2) a failure to communicate and effectively alert former patients about the adverse effects. See Bavli and Steel, *supra* note 16.

[120] See, for example, unethical experiments on African Americans: A. M. Brandt , “Racism and Research: The Case of the Tuskegee Syphilis Study” The Hastings Center 8, no. 6 (1978): 21–29; S. L. Smith, “Mustard Gas and American Race-Based Human Experimentation in World War II,” *Journal of Law, Medicine & Ethics* 36, no. 3 (2008): 517–21; D. P. Scharf et al., “More than Tuskegee: Understanding Mistrust about Research Participation,” *Journal of Health Care for the Poor and Underserved* 21, no. 3 (2010): 879–897.

[121] This could be done by, for example, allowing members of disadvantage communities experiencing the harm to participate in research about the adverse effects of error, or encouraging community leaders to communicate with group members about the risks.

[122] N. Davidovitch and A. Margalit , “Public Health, Racial Tensions, and Body Politic: Mass Ringworm Irradiation in Israel, 1949-1960,” Journal of Law, Medicine & Ethics 36, no. 3 (2008): 522–529. 10.1111/j.1748-720X.2008.300.x 18840245

[123] Bavli and Steel, *supra* note 16.

[124] Centers for Disease Control and Prevention, “Opioid Data Analysis and Resources” (2020), *available at* <https://www.cdc.gov> (last visited October 6, 2021).

[125] Public Health Agency of Canada, *Apparent Opioid and Stimulant Toxicity Deaths*, (Ottawa, 2021), *avaialbe at* <https://publications.gc.ca/collections/collection_2021/aspc-phac/HP33-3-2020-eng-3.pdf> (last visited October 6, 2021).

[126] For discussions on the history of opioids and addiction, See K. Wailoo , Pain: A Political History (Baltimore: Johns Hopkins Press, 2014); D. Herzberg, *White Market Drugs: Big Pharma and the Hidden History of Addiction in America* (Chicago and London: The University of Chicago Press, 2020).

[127] I. A. Dhalla , et al., “Facing up to the Prescription Opioid Crisis,” BMJ 343 (2011): d5142; J. Lexchin and J. C. Kohler,“The Danger of Imperfect Regulation: OxyContin Use in the United States and Canada,” *International Journal of Risk and Safety in Medicine* 23, no. 4 (2011): 233–240. 22156088 10.3233/JRS-2011-0539

[128] N. B. King et al., “Determinants of Increased Opioid-Related Mortality in the United States and Canada, 1990-2013: A Systematic Review,” American Journal of Public Health 104, no. 8 (2014): e32–e42; A. Lembke, *Drug Dealer, MD: How Doctors Were Duped, Patients Got Hooked, and Why It’s So Hard to Stop* (Baltimore: JHU Press, 2016); M. A. Makary, et al., “Overprescribing Is Major Contributor to Opioid Crisis,” *BMJ* 359 (2017): 19–20. A working paper by the National Bureau of Economic Research found that states that limited the marketing of OxyContin and its distribution after approval in 1995 had a lower rate of overdose death than states that did not. The report concludes that “the introduction and marketing of OxyContin explain a substantial share of overdose deaths over the last two decades,” See E. A. Alpert et al., “Origins of the Opioid Crisis and its Enduring Impacts,” *National Bureau of Economic Research*, No. w26500 (2019). 10.2105/AJPH.2014.301966 PMC4103240 24922138

[129] Government Accounting Office*. Report to Congressional Requesters: Prescription Drugs: OxyContin Abuse and Diversion and Efforts to Address the Problem*, GAO-04-011 (Washington: December, 2003); B. Meier, *Pain Killer: An Empire of Deceit and the Origin of America’s Opioid Epidemic*, 2nd ed., (New York: Random House, 2018).

[130] See L. E. Chaparro et al., “Opioids Compared with Placebo or Other Treatments for Chronic Low Back Pain: An Update of the Cochrane Review,” Spine 39, no. 7 (2014): 556–563; R. Chou et al., “The Effectiveness and Risks of Long-Term Opioid Therapy for Chronic Pain: A Systematic Review for a National Institutes of Health Pathways to Prevention Workshop,” *Annals of Internal Medicine* 162, no. 4 (2015): 276–286; T. R. Frieden and D. Houry, “Reducing the Risks of Relief—the CDC Opioid-Prescribing Guideline,” *NEJM* 374, no. 16 (2016): 1501–1504; E. E. Krebs et al., “Effect of Opioid vs Nonopioid Medications on Pain-Related Function in Patients with Chronic Back Pain or Hip or Knee Osteoarthritis Pain the SPACE Randomized Clinical Trial,” *JAMA* 319, no. 9 (2018): 872–882; Dhalla, et al., *supra* note 127. 24480962 10.1097/BRS.0000000000000249

[131] Dhalla, et al., *supra* note 127; Lembke, *supra* note 128; Makary et al., *supra* note 128.

[132] Food and Drugs Administration, “Prescription Drug Labeling Resources,” *available at* <https://www.fda.gov/drugs/laws-acts-and-rules/prescription-drug-labeling-resources> (last visted October 7, 2021).

[133] Meier, *supra* note 129.

[134] Dhalla, et al., *supra* note 128; D. N. Juurlink , “Rethinking ‘Doing Well’ on Chronic Opioid Therapy,” Canadian Medical Association Journal 189, no. 39 (2017): E1222–23; A. Kolodny and T. R. Frieden,“Ten Steps the Federal Government Should Take Now to Reverse the Opioid Addiction Epidemic,” *JAMA* 318, no. 16 (2017): 1537–1538. 10.1001/jama.2017.14567 29049522

[135] This approach was echoed by a former sales representative who worked for Purdue and described in an interview with the *New Yorker* that he was trained to quote the label as a key message to persuade physicians that the drug was safe and effective. Specifically, he referenced memorizing and using the phrase “…is believed to reduce the abuse liability…” from the drug’s label in his meeting with physicians, see D. Remnick, “How OxyContin Was Sold to the Masses,” *The New Yorker,* October 27, 2017, *available at <* https://www.newyorker.com/podcast/the-new-yorker-radio-hour/how-oxycontin-was-sold-to-the-masses> (last visited October 7, 2021).

[136] Meier, *supra* note 129

[137] Government Accounting Office, *supra* note 129.

[138] Cite in Meier, *supra* note 129.

[139] Dr. Nathaniel Katz, previously chair of the FDA’s Advisory Committee, Anesthesia, Critical Care, and Addiction Products Division, used much harsher language to describe the agency’s approval of OxyContin. He argued: “Looking back…OxyContin should never have been approved in the absence of better information on the effectiveness and the consequences of narcotics. Why did we take,” he added, “the scraps of inadequate data that we had at that time and allowed ourselves to go so far with allowing shifts in prescribing policy based on such inadequate data?… It occurred to me at that time that we would never tolerate this in relation to other drugs.” Quoted in C. McGreal, *American Overdose: The Opioid Tragedy in Three Acts* (New York: PublicAffairs, 2018).

[140] CBS News. 2019. “Did the FDA Ignite the Opioid Epidemic?” *60 Minutes*, February 24, 2019, *available at* <https://www.cbsnews.com/news/opioid-epidemic-did-the-fda-ignite-the-crisis-60-minutes/> (last visited October 7, 2021).

[141] Quoted in J. Mitchell, “How the FDA Helped Pave the Way for an Opioid Epidemic,” *Mississippi Clarion Ledger*, January 26, 2018, *available at* <https://www.clarionledger.com/story/news/2018/01/26/opioid-epidemic-how-fda-helped-pave-way/950561001/> (last visited October 7, 2021).

[142] Bavli and Steel, *supra* note 39; Bavli, *supra* note 81.

[143] *Id.*

[144] See A. Kolodny , “How FDA Failures Contributed to the Opioid Crisis,” AMA Journal of Ethics 22, no. 8 (2020): 743–750. 10.1001/amajethics.2020.743 32880367

[145] D. Carpenter and D. Moss , “Introduction,” in D. Carpenter and D. Moss , eds., Preventing Capture: Special Interest Influence in Regulation, and How to Limit It (New York: Cambridge University Press, 2014), at 13.

[146] For discussions about “revoloving doors,” see J. Abraham, “The Pharmaceutical Industry as a Political Player,” Lancet 360, no. 9344 (2002): 1498–1502; T. Makkai and J. Braithwaite, “In and Out of Revolving Door: Making Sense of Regulatory Capture,” *Journal of Public Policy* 12, no. 1 (1992): 61–78. 12433532 10.1016/S0140-6736(02)11477-2

[147] See Kolodny, *supra* note 144.

[148] Lexchin, *supra* note 113.

[149] *Id.* at 13.

[150] Bavli and Steel, *supra* note 39; Bavli, *supra* note 81.

[151] See A. Gaffney et al., “Healing an Ailing Pharmaceutical System: Prescription for Reform for United States and Canada,” BMJ 36 (2018): k1039; Lexchin and Kohler, *supra* note 127; R. Moynihan et al., “Pathways to Independence: Towards Producing and Using Trustworthy Evidence,” *BMJ* 367 (2019): l6576. 10.1136/bmj.l6576 31796508

[152] See, for example, S. E. Hadland et al., “Association of Pharmaceutical Industry Marketing of Opioid Products to Physicians With Subsequent Opioid Prescribing,” JAMA Internal Medicine 178, no. 6 (2018): 861–863; P. T. M. Leung et al., “A 1980 Letter on the Risk of Opioid Addiction,” *NEJM* 376, no. 22 (2017): 2194–2195; S. Podolsky et al., “Preying on Prescribers (and Their Patients) — Pharmaceutical Marketing, Iatrogenic Epidemics, and the Sackler Legacy,” *NEJM* 380, no. 19 (2019): 1785–1787; A. Van Zee, “The Promotion and Marketing of Oxycontin: Commercial Triumph, Public Health Tragedy,” *American Journal of Public Health* 99, no. 2 (2009): 221–227.29799955

[153] See M. Angell , The Truth About the Drug Companies: How They Deceive Us and What to Do About It (New York: Random House, 2004); Dumit, *supra* note 84; G. Posner, *Pharma: Greed, Lies, and the Poisoning of America* (New York: Simon & Schuster, 2020); S. Sismondo, *Ghost-Managed Medicine: Big Pharma’s Invisible Hands* (Manchester: Mattering Press, 2018).

[154] F. Godlee , “Covid-19: We Need New Thinking and New Leadership,” BMJ 371 (2020): m4358.

